# Evidence that the stress hormone cortisol regulates biofilm formation differently among *Flavobacterium columnare* isolates

**DOI:** 10.1186/s13567-019-0641-3

**Published:** 2019-04-11

**Authors:** Annelies Maria Declercq, Wenlong Cai, Eber Naranjo, Wilawan Thongda, Venessa Eeckhaut, Eva Bauwens, Covadonga Arias, Leonardo De La Fuente, Benjamin H. Beck, Miles D. Lange, Eric Peatman, Freddy Haesebrouck, Johan Aerts, Annemie Decostere

**Affiliations:** 1 Department of Pathology, Bacteriology and Poultry Diseases, Faculty of Veterinary Medicine, Ghent University, Ghent, 9000 Belgium; 2 Stress Physiology Research Group, Faculty of Pharmaceutical Sciences, Ghent University, Ostend, 8400 Belgium; 3 School of Fisheries, Aquaculture, and Aquatic Sciences, Aquatic Microbiology Laboratory, Auburn University, Auburn, AL 36849 USA; 4 Department of Entomology and Plant Pathology, Auburn University, Auburn, AL 36849 USA; 5 School of Fisheries, Aquaculture, and Aquatic Sciences, Aquatic Genetics and Genomics, Auburn University, Auburn, AL 36849 USA; 6 United States Department of Agriculture, Agricultural Research Service, Aquatic Animal Health Research Unit, Auburn, AL 36849 USA; 7 United States Department of Agriculture, Agricultural Research Service, Harry K. Dupree Stuttgart National Aquaculture Research Center, Stuttgart, AR 72160 USA; 8 Stress Physiology Research Group, Animal Sciences Unit, Flanders Research Institute for Agriculture, Fisheries and Food, Ostend, 8400 Belgium

## Abstract

The impact of cortisol on *Flavobacterium columnare* biofilm formation was explored. Firstly, the dynamics of biofilm formation by one highly (HV) and one low virulent (LV) *F. columnare* isolate with and without the stress hormone cortisol under microfluidic flow conditions was characterized. This to confirm that *F. columnare* cells could form biofilm under cortisol supplementation, and to compare the temporal and structural differences between different treatment groups. One trial revealed that in both isolates cell aggregates resembling biofilms occurred within 7-h post-inoculation. Consequently, cell clusters were sloughed away, followed by a rebuilding of bacterial cell aggregates, suggestive for a high spreading capacity. While the HV isolate revealed cell aggregates formed upstream at all time-points, for the LV isolate this was only seen upon cortisol supplementation. Secondly, the transcriptional effect of genes (*gld*K*, gld*L*, gld*M, *gld*N, *spr*A, *spr*E, *spr*T, and *por*V) belonging to the Type IX secretion system involved in gliding motility was investigated in planktonic and biofilm cells of a HV and LV isolate to which no, a low (LD) or high (HD) dose of cortisol was added. Significantly lower expression of gliding genes *gld*K, *gld*L*, gld*M and *gld*N, and of protein secretion regulator *por*V was seen in the LV isolate planktonic cells supplemented with a HD-cortisol. The LV isolate biofilm cells treated with the HD-cortisol showed a significant upregulation of *spr*T, encoding mobile surface adhesion important in bacterial colonization. This is the first evidence for the co-regulatory effect of cortisol on biofilm formation and *F. columnare* gliding gene expression.

**Electronic supplementary material:**

The online version of this article (10.1186/s13567-019-0641-3) contains supplementary material, which is available to authorized users.

## Introduction

Living organisms survive by maintaining an equilibrium, or homeostasis, that is continuously challenged by intrinsic or extrinsic disturbing stimuli [1]. The latter are called stressors, and are defined as stimuli that trigger a stress response [2]. Fish that endure stress react by initiating an endocrine stress response through the activation of the hypothalamic-pituitary-interrenal axis, which results in the release of glucocorticoids into the blood, in particular cortisol in teleost fish [3, 4]. Cortisol exerts effects at both the blood and tissue level [4], and is further excreted via the mucus, urine, and faeces [5]. Stress is not necessarily detrimental to fish [3]. A distinction should be made between stressors that stimulate the animal (“eustress”), and those that may result in a chronic, pathological state (“distress”) [4]. Indeed, if a stressor persists over time, or when too intensive, the stress response may lose its adaptive value and become dysfunctional [2, 4].

For the majority of bacterial fish pathogens, chronic stress is considered a key factor in disease outbreaks as is the case for *Flavobacterium columnare* (*F. columnare*), the causative agent of columnaris disease [6, 7]. This disease can give rise to multiple clinical signs, such as skin lesions, mouth rot, and gill lesions [7] and causes huge financial losses in carp, catfish and trout aquaculture [6, 8–10]. A consistent finding throughout the challenges performed by Declercq et al. [11, 12] was the marked individual variation in susceptibility to columnaris disease, especially for the fish exposed to the low virulent isolate. Clinically healthy fish were co-present with moribund animals displaying severe gill pathology following inoculation with a low virulent *F. columnare* isolate. Currently, a thorough explanation for these results is being debated. Individual coping styles of fish have been attributed to varying cortisol levels in fish following an acute stress stimulus [13, 14]. In the aforementioned immersion challenges to reproduce columnaris disease, netting the fish and placing them in the inoculation bath for 90 min were shown to be stress stimuli. The challenge procedure itself may have resulted in fish displaying varying plasma and mucus cortisol levels engendering different degrees of susceptibility towards the different *F. columnare* isolates with which the fish were exposed. To validate this line of reasoning, one may propose there is a direct effect of cortisol on the *F. columnare* bacterial cells. Indeed, a recent study from our lab revealed a dose-dependent impact of cortisol on the colony morphology of *F. columnare* [15]. The higher the cortisol concentration in the bacterial cultures, the less rhizoid the resulting colonies were [15]. Gliding is a well-described trait for *F. columnare* cells [6, 16, 17] and is reflected in the rhizoid shape of colonies, a feature positively linked to virulence [18]. The type IX secretion system (T9SS) is required for gliding motility as it is involved in the secretion of cell surface proteins of the gliding apparatus [19–22]. Genetic analyses suggest that GldK, GldL, GldM, GldN, SprA, SprE, SprT, and PorV are T9SS components [21, 22]. This system is needed for the secretion of SprB and RemA, mobile surface adhesins involved in gliding and movement over surfaces. Cells with mutations in the genes encoding these adhesins were affected in gliding [23] and failed to attach to glass [21].

The formation of biofilms on gill filaments is an important step in establishing gill pathology in columnaris disease as observed during in vivo trials [12]. Biofilm formation is a multistage process that is initiated through different stimuli with free-living or planktonic bacterial cells gliding over a surface prior to attachment [24–26]. The cells lose their motility and cluster together [27] and are then referred to as sessile or biofilm cells. Bearing in mind the above cited effects of cortisol on the rhizoid shape of the bacterial colonies and their gliding motility, one might expect that the presence of cortisol may impact the capacity to form biofilms. Likewise, differential expression of gliding motility genes may be presumed in biofilm-forming cells versus their free-swimming counterparts following incubation in the presence and/or absence of cortisol.

In this framework, the objective of the present study was to evaluate whether cortisol has an impact on *F. columnare* biofilm formation among a low (LV) and highly virulent (HV) carp (*Cyprinus carpio*) isolate, with special interest in the effect on the LV isolate. Biofilm formation was evaluated under conditions of flow by using microfluidic chambers as previously described [27, 28]. Although the complex operational control, this set-up provided important advantages such as real-time analysis and ability to perform perfusion culture by injecting bacteria into an environment under continuous flow. As loss of *F. columnare* gliding is important to initiate biofilm formation [27] the impact of cortisol on the expression of genes (*gld*K, *gld*L, *gld*M and *gld*N, *spr*A, *spr*E, *spr*T, and *por*V) involved in motility, adhesion and protein secretion of Flavobacteriaceae was evaluated. This was done in biofilm-forming versus planktonic cells of a low and highly virulent *F. columnare* isolate to which no, a low or a high dose of cortisol was added. This study is the first to investigate whether cortisol directly steers the gliding capacity in *F. columnare* on a genetic level and therefore impacts biofilm formation, hence providing new insights into *F. columnare* virulence mechanisms.

## Materials and methods

### *F. columnare* culture conditions

*Flavobacterium columnare* isolates 0901393 and CDI-A recovered from carp were used in this study. Their virulence profiles were previously determined [11, 12]. Isolates 0901393 and CDI-A have been shown to be highly virulent (HV) and low virulent (LV), respectively, in laboratory challenges. Both isolates are genomovar I, as determined by the Aquatic Microbiology Laboratory of Auburn University (Alabama, USA) using 16S-restriction fragment length polymorphism protocol [29].

The isolates were grown in triplicate for 36 h at 28 °C on modified Shieh agar plates. For each of the three plates per isolate, five random colonies (all colonies grown showed homogenous sizes and rhizoid shapes) per plate were transferred into 15 mL Falcon tubes filled with 4 mL of modified Shieh broth, which were shaken overnight at 28 °C and 150 rpm. Of the cultivated broths, 1 mL was added to 150 mL Erlenmeyer flasks filled with 99 mL of modified Shieh broth (1/100 dilution). Cortisol (hydrocortisone-water soluble, Sigma Aldrich, Overijse, Belgium) was dissolved in ultrapure water, filtered through a 0.2 µm filter (Millipore, Bedford, USA) and diluted to obtain a final concentration in the broth culture of 500 µg/L (high dose; HD) and 50 µg/L (low dose; LD) accounting for 1.378 µM and 0.138 µM cortisol, respectively. For each isolate, the HD and LD of cortisol was added and a non-treated control with only sterile ultrapure water were tested in triplicate (biological replicates). Flasks were incubated at 28 °C for 24 h at 150 rpm. One mL of each tube was collected to measure optical densities (OD). The OD_600_ of the 24 h cultured broths was 0.45 ± 0.05.

For the microfluidic chamber experiments, one mL of one HD cortisol and control cultivated broth cultures of the HV and LV isolates was transferred into a 1 mL syringe and coupled to an automated syringe pump and injected into a microfluidic chamber as described below.

For the gene expression study, both planktonic and biofilm cells were harvested from each cultivated HD or LD cortisol, and control broth cultures (hence triplicates) of the HV and LV isolates. For the planktonic cells, 99 mL of each cultivated broth was divided equally over three 50 mL Falcon tubes and centrifuged (4 °C, 4750 *g*) for 20 min. Biofilm cells were scraped from the edge of the Erlenmeyer flask with a cell scraper (Greiner Bio One, Vilvoorde, Belgium). Pellets from planktonic and biofilm cells were transferred to a 1.5 mL Eppendorf tube, after which 500 µL RNA later (Ambion^®^ Thermo Fisher Scientific) was added. Samples were stored at 4 °C for 24 h, after which they were frozen at −20 °C until analysis.

### Microfluidic chambers

Biofilm formation under flow conditions was evaluated by using microfluidic chambers [27, 28] which were composed of two parallel microchannels (80 μm wide by 3.7 cm long by 50 μm deep). The chamber consisted of a molded polydimethylsiloxane body, attached to a cover slip and a supporting glass microscope slide. Each channel had two inlets to allow the separate entry of media and bacteria, and an outlet to allow media and bacteria to flow out via the other end. The channels were completely filled with fluid and the flows in the media inlets were started simultaneously to avoid air bubbles in the tubing during the experiment. Modified Shieh medium was collected in 10 mL syringes which were coupled via an automated syringe pump to the media inlet. The flow of the modified Shieh medium inside the chamber was kept constant (10 μL/min) for the duration of each experiment. In the first experiment, the HV and LV isolates growth was compared in parallel at room temperature (24 °C) for 24 h to verify whether biofilm was formed and if so, what qualitative differences in biofilm formation between both isolates could be observed. Cell suspensions were introduced into each channel via the bacterial inlet at 1 µL/min for 1 h, after which bacterial inflow was stopped. In a second trial, for each isolate, parallel comparisons were made between the control and HD of cortisol cultivated in broth. The flow rate of the bacteria was kept constant at 1 µL/min for the entire duration of the experiment (12 h) to prevent the chambers from clogging. Each microfluidic experiment was performed in singlefold merely to confirm that *F. columnare* cells could indeed form biofilm under cortisol supplementation, and to compare the temporal and structural differences between the different treatment groups. Microfluidic chambers were mounted onto a Nikon Eclipse Ti inverted microscope (Nikon, Melville, NY, USA) and observed at 40× with phase-contrast optics to monitor cell aggregation and biofilm formation for the duration of each experiment. Images were captured every 30 s by using a Nikon DS-Q1 digital camera (Nikon) controlled by NIS-Elements software (Nikon).

### Differential gene expression studies between biofilm and planktonic cells

#### RNA extraction and RT-qPCR

The bacterial cells were centrifuged (5000 *g*, 20 min) and the RNA later was removed from the samples, after which 1 mL TRIzol™ (Invitrogen Thermo Fisher Scientific) was added. Subsequently, the samples were homogenized by pipetting up and down. Samples containing twelve 2.3 mm silica beads were placed in the Tissue Lyser (Qiagen, Antwerp, Belgium) three times for 45 s at 20 Hz, with one min between each cycle during which the samples were preserved on ice. Samples were then centrifuged for 5 min at 4 °C and 12 000 *g* and the supernatant was transferred to a new Eppendorf tube. After five min incubation at room temperature (RT), 0.2 mL chloroform (Sigma Aldrich, Overijse, Belgium) was added per 1 mL TRIzol™. After another incubation period of three min at RT, the samples were centrifuged for 30 min at 4 °C and 12 000 *g*. The colorless, upper aqueous phase was transferred to a new Eppendorf tube and one volume of 100% ethanol (VWR, Oud-Heverlee, Belgium) was added. The content of the tube was then loaded onto an RNeasy spin column (RNeasy Mini Kit, Qiagen), according to manufacturer’s protocol. The flow-through was discarded. Thirty µL RNase-free water was added directly to the spin membrane and centrifuged for one min at 8000 *g* to elute the RNA. The flow-through contained the total RNA and residual genomic DNA was removed with DNA-free™ (Ambion^®^ Thermo Fisher Scientific) according to the manufacturer’s instructions. RNA concentration was measured with a NanoDrop^®^ ND-1000 Spectrophotometer.

High-quality RNA samples were used to initiate first strand cDNA synthesis. One µg of DNAse-treated RNA was used as template in 20 µL cDNA synthesis reactions applying iScript™cDNA Synthesis Kit (Bio-Rad, Temse, Belgium). cDNA synthesis reactions were incubated for five min at 25 °C, 20 min at 46 °C, and 1 min at 95 °C. Each cDNA sample was then diluted 1/10. Afterwards, 10 µL of each cDNA sample was pooled to set up a twofold dilution standard curve. Each cDNA sample was run in triplicate using a CFX384™ RT-PCR System or a CFX96™ RT-PCR System with a C1000 Thermal Cycler (Bio-Rad, Hercules, CA, USA). The reaction mixture of 12 μL consisted of 6 μL of iQ™ SYBR^®^ Green Supermix (Bio-Rad), 3.95 μL of high pressure liquid chromatography water (Merck Millipore, Overijse, Belgium), 0.025 μL of each primer (0.05 µM), and 2 μL cDNA. The amplification conditions were as follows: 95 °C for 3 min, followed by 40 cycles of 95 °C for 10 s and 60 °C as melting temperature for 30 s. Intra-run variability was ascertained by comparing the obtained Cq-values for the standard twofold dilution series of cDNA within one RT-PCR run, whereas inter-run variability was evaluated by comparing those values between three RT-PCR runs (see further). The amplification efficiency and R^2^ were calculated using the Bio-Rad CFX Manager v1.6 software (Bio-Rad) (Table 1).

**Table 1:** **Primer sequences and amplicon details used in RT-qPCR assay for** ***Flavobacterium columnare***

Primer name	Sequence (5′–3′)	Amplicon length (bp)	Efficiency (%)	R^2^ (%)	References
Primers for target genes (gld-genes are involved in motility, spr-genes in adhesion and porV in virulence)					
FC_gldK_fwd	GCCAAATAGCACCCTCTATCA	102	103.0	98.9	This study
FC_gldK_rev	AACAGAAGCCGAATGGGAATA				
FC_gldL_fwd	GCTTCTGTACCTAAACCAGCA	94	99.9	99.5	This study
FC_gldL_rev	TTGGTGCGGCAGTAGTAATC				
FC_gldM_fwd	ACTACCACATCACCTCTTTGTG	93	102.4	99.2	This study
FC_gldM_rev	TCCAGGGCAACCAACAATAG				
FC_gldN_fwd	GCAAGCGCTATGCTTATTGCTGGT	131	97.8	99.0	[31]
FC_gldN_rev	GCAGTTGGTTGTCCCCCTGCT				
FC_sprA_fwd	AGGCGATGGTATTTCGTTAGG	122	101.9	99.5	This study
FC_sprA_rev	GTACGCGTCCTGCTTGATAA				
FC_sprE_fwd	AGCCGTGCAGAAGATAAAGC	151	92.4	99.0	[31]
FC_sprE_rev	ACGCTTCTAATGCGGGTACAA				
FC_sprT_fwd	AACCAGGACTGCATTACGGA	144	91.1	99.2	[31]
FC_sprT_rev	GCTTGATGTTACCTGTGCGTT				
FC_porV_fwd	GTGCCAACTCCTAAAACAGCC	152	102.7	99.3	[31]
FC_porV_rev	AAACCTCCTGGAGCATCACC				
Primers for reference genes					
FC_16S_fwd	ACGATCAAACGGCCATTG	119	105.0	99.4	[30]
FC_16S_rev	AGTAACCTGCCTTCGCAATC				
FC_gap1_fwd	ACCATCCCAAACAGGAGCCGC	98	103.3	99.3	[30]
FC_gap1_rev	CGTCTGCTGTAGGTACGCGCA				
FC_glyA_fwd	CCAAACCCTTGGGGCTATACAACCC	98	103.2	99.6	[30]
FC_glyA_rev	AGAGGGCCTCCTTGATTACCTGGAA				
FC_rplQ_fwd	AGCTGCTAAAGTAGGTGACCGTCC	75	96.7	99.1	[30]
FC_rplQ_rev	GCGTTATCTCCTAAACGGTTCCCCA				

Amplicon length, efficiency, and R^2^ are given for a primer pair consisting of a forward (fwd) and a reverse (rev) primer.

#### qPCR primer design and validation

Primers for amplifying the reference genes were chosen based on previous work by Penttinen et al. [30], and those from the genes of interest based on Penttinen et al. [31] or were designed for use in this study. IDT PrimerQuest Tool [32] was used to design own primer pairs for target genes. The sequences of the primers used in this study are listed in Table 1. The preliminary expression stability testing was first performed with four reference gene candidates (*16S rRNA; gap; glyA; rplQ*) using a mixture of all test samples (*N* = 36) that included samples from planktonic and biofilm cells collected from *F. columnare* cultured in modified Shieh broth to which no, a LD, or a HD of cortisol was added. The data was checked for normality and the expression stabilities were determined via qBase and geNorm and calculated per *F. columnare* isolate. According to these methods, *gap* and *glyA* possessed the best stabilities and were chosen to be used as reference genes in the expression analysis of the dataset of each *F. columnare* isolate. Furthermore, DNAse treated RNA samples were pooled and included in the RT-qPCR assays to assure no hybridization of cDNA to the RNA occurred. A no template control was also included.

#### Statistics

All parameters were statistically modeled using Graphpad Prism 7 with cortisol concentration (0, 50, or 500 µg/L), and cell type (planktonic, or biofilm) as fixed effects. The modelled outcomes were the qPCR-data of the RNA-expression of each of the target genes (*gld*K, *gld*L, *gld*M and *gld*N, *spr*A, *spr*E, *spr*T, and *por*V) and the two final reference genes (*gly*A and *gap*). Expression levels were normalized to the expression of reference genes. The data were analyzed using Wilcoxon matched-pairs signed rank test to compare treatments with the control data within each cell type. Differences were considered to be significant when *p*-values were lower than 0.05.

## Results

### Microfluidic chambers

**Figure 1:**
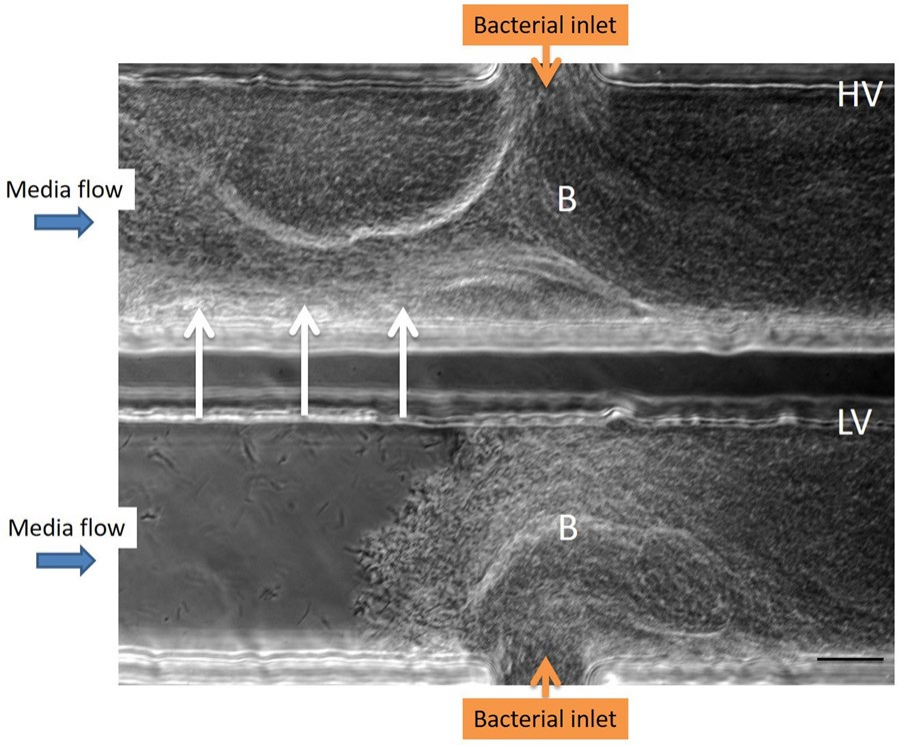
**Biofilm formation of** ***F. columnare*** **isolates in a microfluidic chamber.** Biofilm formation (B) of the highly virulent (HV, upper channel) and low virulent (LV, lower channel) *F. columnare* isolate are compared in parallel at 24 h pi. Bacterial cell suspensions were introduced into each channel via the bacterial inlet at 1 µL/min for 1 h, after which bacterial inflow was stopped. For the HV isolate, bacterial cell clusters completely fill the channel and are even encountered upstream (arrowheads), while the latter is not observed for the LV isolate. This microfluidic experiment was performed in singlefold merely to grasp whether both *F. columnare* isolates could form biofilm formation in the microfluidic chambers. Scale bar 20 µm.

The cells of both the HV and LV isolates clustered rapidly within 4 h post-inoculation (pi), at the bacterial inlet side of the channel. Cell aggregates resembling biofilm filled the entire channel within 7 h pi (see Additional file 1), starting at the bacterial inlet and building up to the middle to eventually reach the opposite side of the channel. Consequently, cell clusters were sloughed away, whereby the channels were cleared, immediately followed by a rebuild of bacterial cell aggregation. At the end of the observation period, the bacterial cells of the HV isolate also formed cell aggregates upstream of the bacterial inlet. For the LV isolate, bacterial cell cluster formation was only seen downstream in the channel (Figure 1).

**Figure 2:**
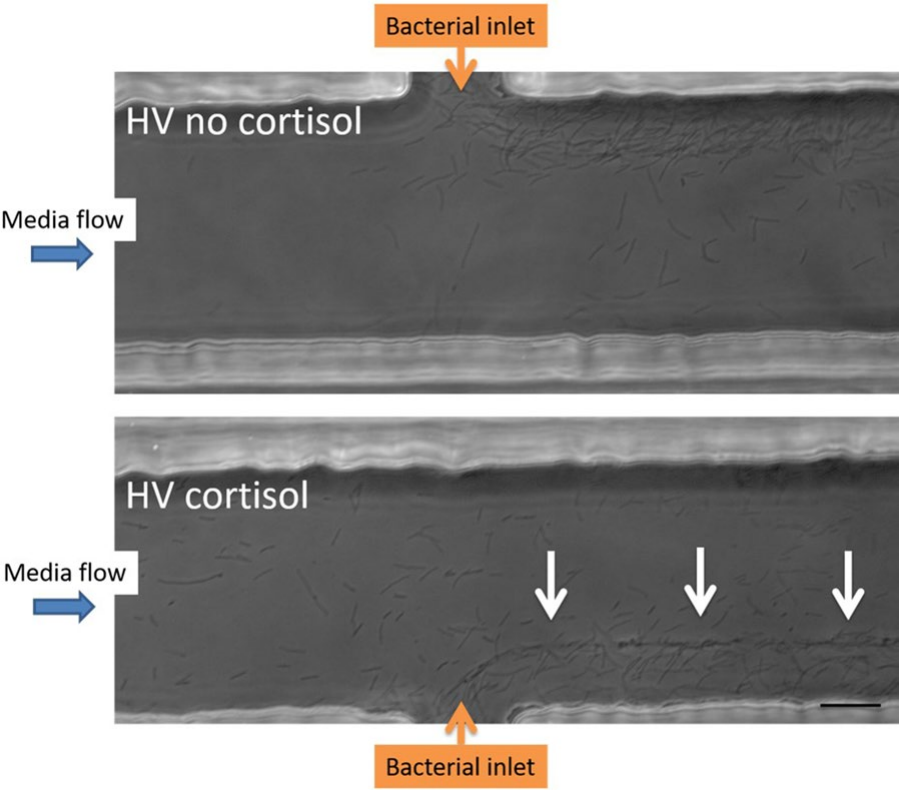
**Biofilm formation of HV** ***F. columnare*** **isolate stimulated with cortisol.** Biofilm formation of the highly virulent (HV) isolate cultivated without (upper channel) and with (lower channel) a high dose of cortisol is compared in parallel at 4 h pi. The flow rate of the bacteria was kept constant at 1 µL/min for the entire duration of the experiment (12 h) to prevent the chambers from clogging. The cortisol supplemented bacterial cells display a more organized cell clustering lining-up at one-third of the microfluidic chamber (white arrows). As the bacterial inflow remained constant during the whole trial to prevent the chamber from clogging, the thickness of biofilm formation was smaller compared to the one formed in the first experiment (Figure 1) in which the complete channel was filled. This microfluidic experiment was performed in singlefold merely to grasp whether the HV *F. columnare* isolate supplemented with and without cortisol could for biofilm formation in the microfluidic chambers. Scale bar 20 µm.

Different patterns of bacterial cell aggregation were observed upon parallel comparison of the HV isolate with no and a HD of cortisol. For the latter, more organized cell aggregates lining up at one-third of the microfluidic chamber were noted, whereas this was not the case for the non-supplemented bacterial cells (Figure 2).

**Figure 3:**
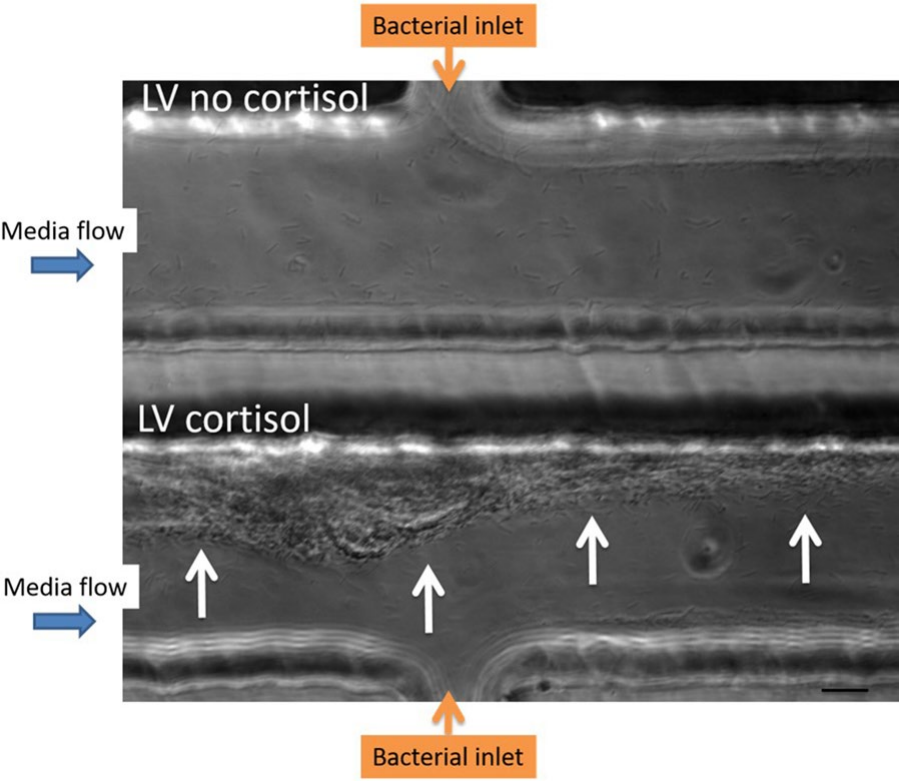
**Biofilm formation of LV** ***F. columnare*** **isolate stimulated with cortisol.** Biofilm formation of the low virulent (LV) isolate cultivated without (upper channel) and with (lower channel) a high dose (HD) of cortisol is compared in parallel at 12 h pi. The flow rate of the bacteria was kept constant at 1 µL/min for the entire duration of the experiment (12 h) to prevent the chambers from clogging. The bacterial cells of the LV isolate with HD of cortisol form aggregates filling the channel up to the middle and moreover biofilm formation upstream from the bacterial inlet (white arrows) is seen, whereas this was not the case for the non-supplemented LV isolate. In the latter, only downstream biofilm formation is seen. As the bacterial inflow remained constant during the whole trial to prevent the chamber from clogging, the thickness of biofilm formation was smaller compared to the one formed in the first experiment (Figure 1) in which the complete channel was filled. This microfluidic experiment was performed in singlefold merely to grasp whether the LV *F. columnare* isolate supplemented with and without cortisol could for biofilm formation in the microfluidic chambers. Scale bar 20 µm.

Parallel comparisons of the LV isolate with no and a HD of cortisol revealed that the latter formed aggregates that filled the channels from the side of bacterial inlet up to the middle of the chamber by the end of the experiment (12 h pi). Moreover, cell clusters were formed upstream from the bacterial inlet (Figure 3), and these phenomena were not observed for the non-supplemented LV isolate.

### Differential gene expression between biofilm and planktonic cells

#### qPCR primer design and validation

The expression stability of each isolate was measured using the qbase and geNorm methods. Hereby, *gap* and *glyA* showed the best stability and were subsequently selected as reference genes used for normalization of the gene expression data. M (mean stability)- and CV (coefficient of variation)-values were averaged resulting 0.88 and 0.30 for the HV isolate and 0.74 and 0.26 for the LV isolate, respectively. All were found to be under the maximum allowed values of 1 and 0.5 for M and CV, respectively.

#### Genetic analyses of genes involved in the T9SS of *F. columnare* grown with various cortisol levels

**Figure 4:**
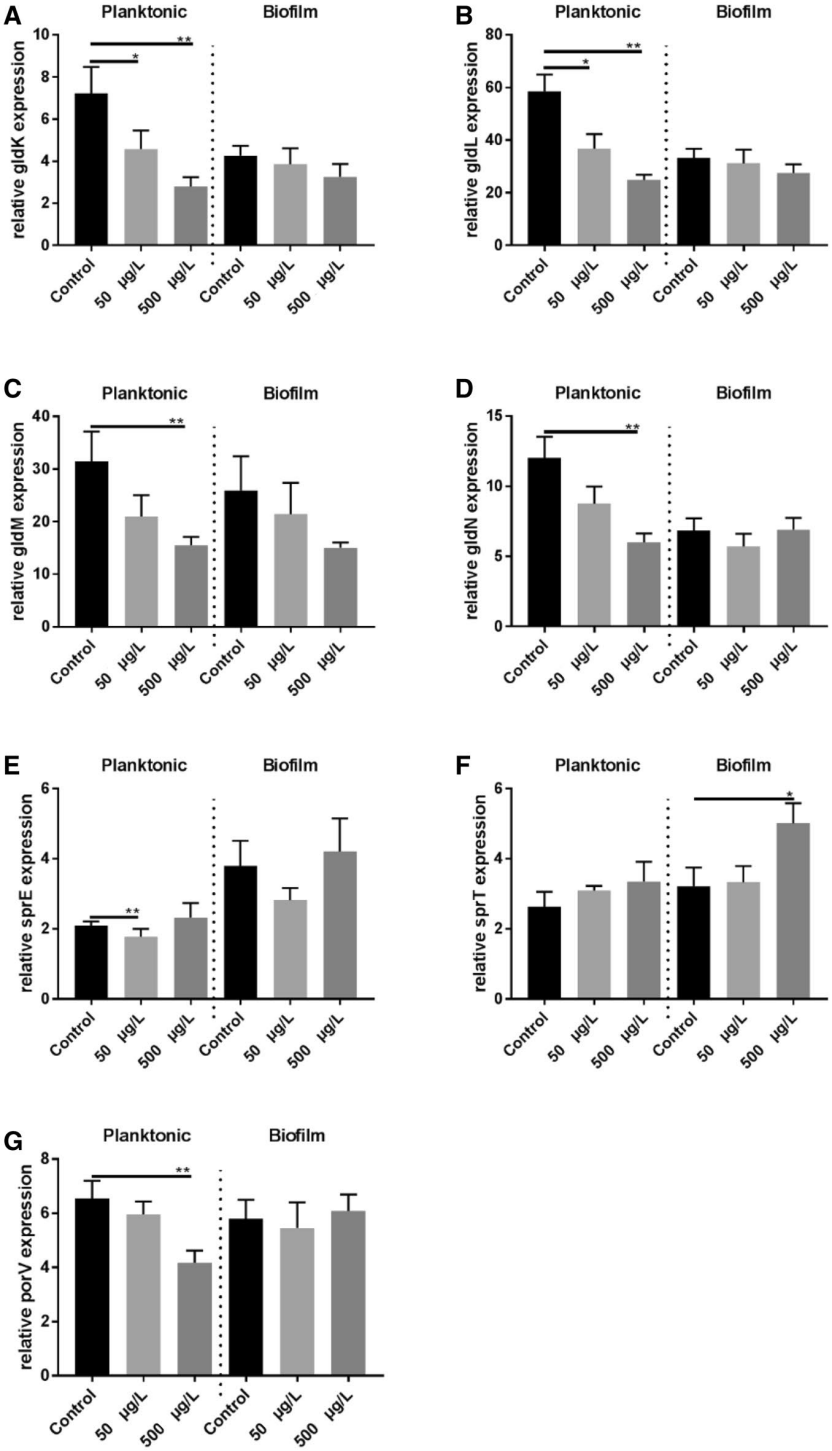
**Mean relative gene expression results ± SEM.** Mean relative gene (*gld*K (**A**), *gld*L (**B**), *gld*M (**C**), *gld*N (**D**), (*spr*E) (**E**), *spr*T (**F**), and *por*V (**G**)) expression results ± SEM in planktonic and biofilm cells of the low (LV) virulent *F. columnare* isolate following supplementation with a low (50 µg/L) or high (500 µg/L) cortisol dose. Only statistically significant differences are presented. The error bars indicate the standard error means and the asterisks indicate significantly different results compared to the non-supplemented controls. Significance levels are indicated on the graphs *, 0.01 ≤ *P* < 0.05; **, 0.001 ≤ *P* < 0.01.

The results of the mean relative gene expression ± SEM are presented in Table 2, Figure 4, and Additional file 2.

**Table 2:** **Mean relative gene expression results ± SEM**

Gene	Isolate	CT	Mean ± SEM	Mean ± SEM	Mean ± SEM500	*p*-value	*p*-value	*p*-value	*p*-value
			0	50		0 vs 50	0 vs 500	P_C_ vs B_C_	P_500_ vs B_500_
*gld*K	LV	P	7.2 ± 1.3	4.6 ± 0.9	2.8 ± 0.5	0.03*	0.004**	0.004**	0.04*
		B	4.3 ± 0.5	3.9 ± 0.8	3.3 ± 0.6	NS	NS		
	HV	P	12.3 ± 1.9	12.5 ± 2.0	8.2 ± 2.2	NS	NS	NS	NS
		B	9.1 ± 2.3	11.3 ± 2.3	9.9 ± 1.0	NS	NS		
*gld*L	LV	P	58.5 ± 6.5	36.9 ± 5.5	24.9 ± 2.0	0.03*	0.004**	0.004**	NS
		B	33.3 ± 3.5	31.2 ± 5.3	27.6 ± 3.3	NS	NS		
	HV	P	75.8 ± 7.8	94.8 ± 10.7	68.2 ± 16.1	NS	NS	0.03*	NS
		B	20.4 ± 8.8	56.7 ± 10.0	46.8 ± 9.6	NS	NS		
*gld*M	LV	P	31.4 ± 5.7	21.0 ± 4.0	15.5 ± 1.5	NS	0.004**	0.03*	NS
		B	25.9 ± 6.5	21.4 ± 6.0	15.0 ± 1.0	NS	NS		
	HV	P	192.3 ± 23.7	283.2 ± 68.0	248.0 ± 40.5	NS	NS	0.047*	NS
		B	59.8 ± 31.9	204.6 ± 37.6	116.1 ± 20.9	NS	NS		
*gld*N	LV	P	12.0 ± 1.5	8.8 ± 1.2	6.0 ± 0.6	NS	0.008**	0.02*	0.008**
		B	6.8 ± 0.9	5.7 ± 0.9	6.9 ± 0.8	NS	NS		
	HV	P	502.2 ± 66.9	552.9 ± 79.6	407.2 ± 89.6	NS	NS	NS	NS
		B	268.8 ± 131.3	313.5 ± 59.1	258.2 ± 59.0	NS	NS		
*spr*A	LV	P	4.2 ± 1.3	3.9 ± 0.9	5.1 ± 1.4	NS	NS	0.004**	NS
		B	17.7 ± 6.1	22.6 ± 7.9	18.0 ± 6.5	NS	NS		
	HV	P	3.5 ± 0.9	3.4 ± 0.3	4.0 ± 0.6	NS	NS	0.03*	0.03*
		B	25.3 ± 4.3	23.3 ± 5.9	18.7 ± 3.5	NS	NS		
*spr*E	LV	P	2.1 ± 0.1	1.8 ± 0.2	2.3 ± 0.4	0.008**	NS	NS	NS
		B	3.8 ± 0.7	2.8 ± 0.4	4.2 ± 0.9	NS	NS		
	HV	P	98.6 ± 18.3	96.0 ± 13.4	124.0 ± 21.6	NS	NS	0.03*	NS
		B	406.6 ± 159.4	149.3 ± 18.4	150.2 ± 51.0	NS	NS		
*spr*T	LV	P	2.6 ± 0.4	3.1 ± 0.1	3.4 ± 0.6	NS	NS	NS	NS
		B	3.2 ± 0.5	3.3 ± 0.5	5.0 ± 0.6	NS	0.04*		
	HV	P	13.9 ± 2.2	13.2 ± 1.4	19.4 ± 1.2	NS	NS	NS	0.04*
		B	37.1 ± 19.2	11.3 ± 2.2	10.0 ± 2.7	NS	NS		
*por*V	LV	P	6.6 ± 0.7	6.0 ± 0.5	4.2 ± 0.4	NS	0.008**	NS	0.008**
		B	5.8 ± 0.7	5.5 ± 0.9	6.1 ± 0.6	NS	NS		
	HV	P	70.6 ± 12.7	80.1 ± 10.3	94.6 ± 17.9	NS	NS	NS	NS
		B	607.2 ± 364.5	63.4 ± 14.7	47.5 ± 15.0	NS	NS		

These include planktonic (P) and biofilm (B) cells of the highly (HV) and low (LV) virulent *F. columnare* isolate to which a cortisol dose of 0 (control), 50 (LD) or 500 µg/L (HD) was added.

0, 50 or 500: added cortisol concentration in µg/L; Mean = mean relative gene expression level; SEM: standard error of means; P_C_ vs B_C_: gene expression in the planktonic cells of the control group compared to the gene expression in the biofilm cells of the control group; P_500_ vs B_500_: gene expression in the planktonic cells treated with 500 µg/L cortisol compared to the gene expression in the biofilm cells treated with 500 µg/L cortisol; significantly different *p*-values are marked in italics with one asterisk indicating *p*-values < 0.05 and two asterisks *p*-values < 0.01; *p*-values depicted are the exact *p*-values obtained after Wilcoxon matched-pairs signed rank test. NS: not significant.

For all *gld*-genes in both the HV and LV isolates a significantly higher expression was found in the planktonic cells compared to their biofilm counterparts in the control broth, except for *gld*K and *gld*N in the HV isolate. When comparing planktonic and biofilm cells incubated in the presence of a HD cortisol (Table 2), this could not be observed.

A significant downregulation was noted for *spr*A (HV + LV isolate), and *spr*E (HV isolate) in the untreated planktonic cells in comparison with the untreated biofilm cells. For the planktonic cells supplemented with a HD cortisol, a significant downregulation of *spr*A (HV isolate), and upregulation of *sprT* (HV isolate) was observed (Table 2).

In planktonic cells of the LV isolate a HD cortisol resulted in a significant downregulation of *gld*K, *gld*L*, gld*M, *gld*N and *por*V while a LD cortisol significantly downregulated *spr*E, *gld*K, and *gld*L compared to the planktonic control cells to which no cortisol was added. A significant inverse relationship for *gld*K and *gld*L was observed between cortisol concentration and gene expression level, with a HD cortisol resulting in more downregulation. In regard to the biofilm cells of the LV isolate incubated in the presence of a HD cortisol, a significant upregulation of *spr*T was observed.

With regard to the HV isolate, no other significant differences in gene expression levels were encountered.

## Discussion

Biofilm formation on the fish’s skin and gills is a well-known feature in fish succumbing to columnaris disease [11, 12]. A more profound knowledge of the biofilm formation and how it interplays with the environment and the host is pivotal to better understand and subsequently mitigate and treat this disease. The pertinent literature describes a plethora of techniques to study biofilm biomass, viability, structure, composition and physiology [25]. For *F. columnare*, biofilm development is visualized applying glass slides, histology, confocal laser scanning microscopy, and scanning and transmission electron microscopy [11, 12, 27]. Cai et al. [27] were the first to use microfluidic flow chambers to assess differences between *F. columnare* isolates in the ability to attach to glass surfaces and form biofilm under flow conditions. This technique has unprecedented potential due to the generated flow and offers a direct and non-destructive observation of developing biofilms providing continuous flow conditions as could be encountered in the aquatic environment [27]. The advantage is that with this system, the process of biofilm formation can be studied spatially and temporarily in conditions that mimic the natural habitats of these bacteria. In the present study, biofilm formation of a HV and LV *F. columnare* isolate was compared in parallel in these microfluidic chambers. Both isolates formed cell aggregates resembling biofilm that filled the complete channel within 7 h pi. This is in contrast to the findings of Cai et al. [27], where the HV isolate was able to develop microcolonies completely colonizing the channel whereas the LV isolate remained confined to the sides. We speculate that the different phenomena of the biofilm development were due to strain or physicochemical variations in the test. Strains of different genomovars have exhibited great genetic heterogeneity [33, 34] and the response to physiochemical factors has proven to be significantly different among different isolates [27]. In this study, only in the channel filled with the HV isolate, bacterial fragments were shown to detach and disperse several times during the 24 h observation period. This phenomenon occurred only once in the channel of the LV isolate. The building up of cell aggregates, detaching, and rebuilding again, is indicative for the spreading capacity of HV *F. columnare* cells. The tendency of individual microcolonies containing biofilm cells to break off when their tensile strength is exceeded, is reported to have the greatest impact on the outcome of (persisting) bacterial infections [35] and permits bacterial cells to escape the confines of the biofilm and colonize new areas [36]. Though caution is needed when extrapolating results of the microfluidic chambers as the experiments were not repeated in time, these new findings might explain results encountered in former in vivo trials where the HV isolate induced gill lesions throughout the entire gill, while for the majority of carp challenged with the LV isolate colonization halted at the filament middle section [11, 12].

Important criteria to incorporate in the development of a model biofilm system are the media flow rate and shear forces [33]. Shear stress suppresses the development of a biofilm matrix but provides more opportunities for new biofilm formation [37]. The latter would occur by improving motility [37], and providing more opportunities for bacterial cells to adhere and disperse [38]. A balance between the two opposing effects may be needed to provide optimal shear stress conditions for promoting biofilm growth [37]. In this study, a continuous high media flow was chosen according to previous studies [27, 39]. The chamber created lower shear forces at the sides of the channels, with the maximum shear force at the middle of the channel [27]. Moreover, a high flow rate prevents the chambers from clogging over time, which has been mentioned as a limitation of microfluidic channels [25].

The cortisol doses added to the bacterial broths in the gene expression trials were 50 (LD) and 500 µg/L (HD), respectively. One may raise the question via what tissues bacterial cells come in contact with this stress hormone. The first matrix *F. columnare* encounters upon adhering to the gill tissue, is the mucus. As far as we know, studies measuring cortisol levels in mucus of fish are confined to those of Guardiola et al. [40]. The latter research group compared the cortisol levels retrieved in serum and skin mucus of gilthead seabream (*Sparus aurata* L.) and surrounding water after acute crowding of the fish. Two hours after onset of the acute stress, only plasma cortisol levels (345.74 ± 23.99 µg/L) were significantly higher compared to unstressed control animals. At 24 h after exposure, plasma cortisol levels decreased and were no longer significantly different from those of the control animals, while skin mucus and water cortisol levels were significantly elevated compared to the controls with levels of 111.98 ± 9.22 and 29.02 ± 4.61 µg/L, respectively. However, one must take into account that the concentration level of excreted non-metabolized cortisol is just a minor fraction of the total glucocorticoids present in the water and on the mucus. Subsequently, one could argue whether these values were a sum of glucocorticoids instead of just cortisol as the antibody based enzymatic immunoassay is biased by cross-reactivity making in-depth analytical validation of utmost importance. As a consequence, extrapolation of these findings to gill mucus remains to be elucidated. Noteworthy is that, already at 4 h post-challenge with a HV isolate, gill filament destruction is evident resulting in *F. columnare* bacterial cells coming into direct contact with plasma, which hence may be a second manner whereby the bacterial cells may be exposed to cortisol. Plasma, the most used matrix for quantification of acute stress across vertebrate species, is known to show a variety of cortisol levels ranging from low (e.g. from circadian rhythmicity) to extremely high (e.g. from severe, uncontrollable, intense, unpredictable stressor). Based on this broad range of plasma cortisol values, the tested concentrations were selected. Hereby, HD (500 µg/L) was chosen to mimic plasma cortisol levels retrieved from carp blood following an acute stressor, as for example Bertotto et al. [41] found average values of 396.8 ± 192.3 μg/L while unstressed animals revealing plasma cortisol levels of 80.7 ± 42.6 µg/L (*P* < 0.01). The HD cortisol hereby ensuring an initial glance on a “physiological” level of cortisol, while the LD cortisol representing plasma cortisol levels of unstressed control individuals.

As mentioned, to enable *F. columnare* cells to form microcolonies on glass slides, gliding capacity is lost [27]. At this point, they are referred to as sessile or biofilm cells. A significant downregulation of the gliding genes *gld*K, *gld*L*, gld*M and *gld*N was seen in the planktonic cells upon incubating the LV isolate culture in the presence of cortisol. Moreover, *gld*K and *gld*L showed a significant inverse relationship between cortisol concentrations and gene expression levels, with higher cortisol doses resulting in less gene expression, suggesting a dose–response effect. In *F. johnsoniae*, *gld*K, *gld*L*, gld*M and *gld*N form an operon [21] and the proteins encoded by these genes interact to form part of the *F. johnsoniae* T9SS. Cells with mutations in these genes, grow well as wild-type cells but are completely non-motile [23]. In *F. columnare* in particular, gene mutation in *gld*N results in bacteria that lack gliding motility [22]. Hence, one might expect that a downregulation of these genes will decrease motility, possibly urging the planktonic cells to adhere and form biofilms. The finding that the expression of the *gld*-genes was significantly lower in the biofilm cells compared to their planktonic counterparts, endorses the above stated hypothesis. This was also encountered in *F. psychrophilum*, in which motility-related genes (amongst which *gld*J, *gld*N, *gld*M, and *gld*L) were significantly downregulated in the biofilm state [42]. This phenomenon was no longer encountered upon comparing planktonic and biofilm cells incubated in the presence of a HD cortisol. Li et al. [22] found that Δ*gld*N mutants exhibited reduced virulence with complementation restoring virulence. Furthermore, in this study, a significant downregulation in *por*V was found in the planktonic cells of the LV isolate treated with the HD cortisol. For *F. columnare*, mutants in this gene exhibited reduced virulence, but retained gliding motility [22]. These findings might suggest that cortisol may also interfere with protein secretion and hence impact virulence. However, further research is imperative to refute or rectify this hypothesis.

Another interesting finding was the significant upregulation of *spr*T in the biofilm cells collected from the LV isolate treated with the HD cortisol. SprT belongs to T9SS [19, 21, 43, 44].

The results of this study support the initial research hypothesis that cortisol, as dominant stress-induced glucocorticoid in teleost fish, exerts a favorable effect on the initial colonization process in the course of disease in the LV *F. columnare* cells. In the latter, a switch from a planktonic to sessile state might be induced by a downregulation in gliding genes, and hence a loss of the gliding characteristic. Once biofilm cells of the LV isolate are formed, the high dose of cortisol might induce an upregulation in genes involved in adhesion and eventual biofilm maturation. The latter matches with the results obtained from the microfluidic chamber experiments in which the LV isolate grown in media supplemented with cortisol, resulted in cell aggregate formation found upstream from the bacterial inlet, referring to adhesion capacities in high shear-environments. This study is the first to demonstrate a direct triggering effect of cortisol on biofilm formation in a bacterial pathogen. This finding again accentuates the need for mitigating stress in aquaculture, thereby not only abolishing a possible impairment of the immune system, but also breaking through a potential direct stimulating effect on biofilm formation by cortisol and possibly other glucocorticoids as commonly encountered in the water.

## Additional files

**Additional file 1.** **Movie of a microfluidic chamber experiment.** The upper chamber shows the highly virulent *F. columnare* cell aggregates resembling biofilm filling the entire channel within 7 h pi, starting at the bacterial inlet and building up to the middle of the channel to eventually reach the opposite side of the channel. The cell aggregates are thick, dense structures, representative of a dense biofilm. The lower chamber shows the build-up of cell aggregates of the low virulent *F. columnare* isolate.

**Additional file 2.** **Mean relative gene expression results ± SEM.** Differential gene *gld*K (A), *gld*L (B), *gld*M (C), *gld*N (D), *por*V (E), *spr*A (F), *spr*E (G), *spr*T (H) expression in planktonic and biofilm cells of highly (HV, isolate 090) virulent *F. columnare* isolates following supplementation with a low (50 µg/L) or high (500 µg/L) cortisol dose. (I) represents the differential *spr*A expression of the low (LV, isolate CDI-A) virulent *F. columnare* isolate. No statistically significant differences were found in the results presented in this graph. The error bars indicate the standard error means.

## Abbreviations

*F. columnare*: *Flavobacterium columnare*
HV: highly virulent
LV: low virulent
HD: high dose
LD: low dose
pi: post-inoculation
M: mean stability
CV: coefficient of variation
