# Supplementary figures and images for: Evidence that the stress hormone cortisol regulates biofilm formation differently among Flavobacterium columnare isolates
Source: Vet Res. 2019 Apr 11;50:24. doi: 10.1186/s13567-019-0641-3 (PMC6458827; doi:10.1186/s13567-019-0641-3)

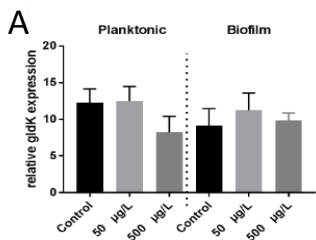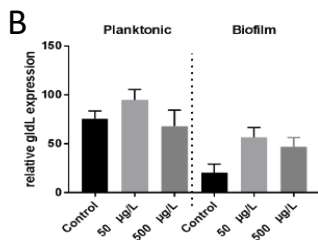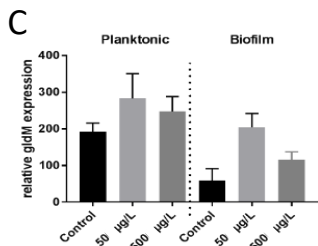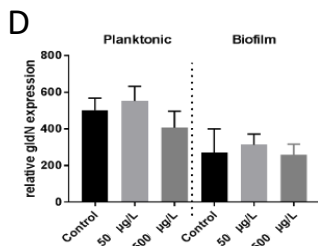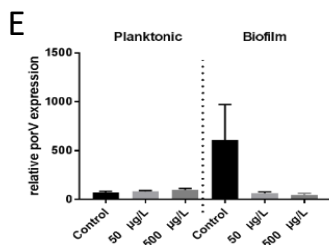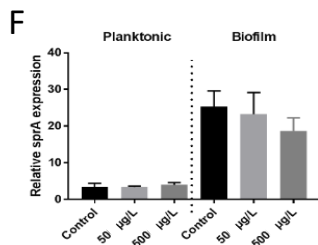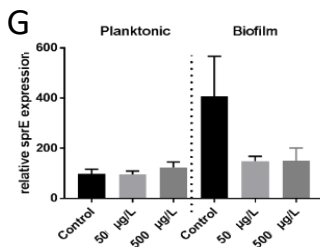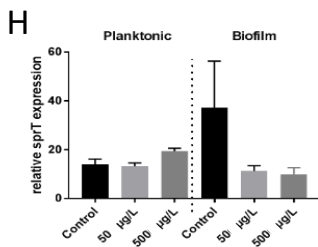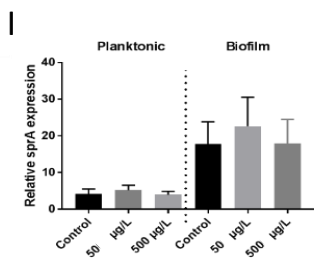

Supplement: Supplementary file 2 — Additional file 2. Mean relative gene expression results ± SEM. Differential gene gldK (A), gldL (B), gldM (C), gldN (D), porV (E), sprA (F), sprE (G), sprT (H) expression in planktonic and biofilm cells of highly (HV, isolate 090) virulent F. columnare isolates following supplementation with a low (50 µg/L) or high (500 µg/L) cortisol dose. (I) represents the differential sprA expression of the low (LV, isolate CDI-A) virulent F. columnare isolate. No statistically significant differences were found in the results presented in this graph. The error bars indicate the standard error means. [file 13567_2019_641_MOESM2_ESM.pdf]
